# Inspired Fluorinated BDD Film for Multifunctional Protection of Downhole Sensor Electrodes

**DOI:** 10.3390/nano15211647

**Published:** 2025-10-28

**Authors:** Jiahao Liu, Shuo Zhao, Jincan Wang, Jiaxi Liu, Xiang Yu, Jing Zhang

**Affiliations:** 1 Programme of Sustainable Energy Technology and Management, Faculty of Science and Technology, Beijing Normal-Hong Kong Baptist University, Zhuhai 519087, China; v530206022@mail.uic.edu.cn; 2 Engineering Research Center, Ministry of Education for Geological Carbon Storage and Low Carbon Utilization of Resources, Beijing Key Laboratory of Materials Utilization of Nonmetallic Minerals and Solid Wastes, School of Materials Science and Technology, China University of Geosciences (Beijing), Beijing 100083, China; shuozhao@buaa.edu.cn (S.Z.); 2103240085@email.cugb.edu.cn (J.W.); 2003250034@email.cugb.edu.cn (J.L.); 3 Institute of Atomic Manufacturing, Beihang University, Beijing 100191, China; 4 Shandong Provincial Key Laboratory of High Strength Lightweight Metallic Materials, Advanced Materials Institute, Qilu University of Technology (Shandong Academy of Sciences), Jinan 250014, China

**Keywords:** conductivity sensor, biomimetic surface, boron-doped diamond, multifunctional coating, downhole protection

## Abstract

Conductivity sensors play a vital role in monitoring production data in oil wells to ensure efficient oilfield operations, and their service performance depends on the durability of Invar alloy electrodes. The alloy electrodes are susceptible to damage from abrasive solid particles, corrosive media, and oil fluids in downhole environments. The degradation of the alloy electrodes directly compromises the signal stability of conductivity sensors, resulting in inaccurate monitoring data. Inspired by the intrinsic oleophobic properties of fish scales, we developed a fluorinated boron-doped diamond (FBDD) film with biomimetic micro–nano structures to enhance the wear resistance, corrosion resistance, and amphiphobicity of Invar alloy electrodes. The fish scale architecture was fabricated through argon-rich hot-filament chemical vapor deposition (90% Ar, 8 h) followed by fluorination. FBDD-coated electrodes surpass industrial benchmarks, exhibiting a friction coefficient of 0.08, wear rate of 5.1 × 10^−7^ mm^3^/(N·mm), corrosion rate of 3.581 × 10^−3^ mm/a, and oil/water contact angles of 95.32°/106.47°. The following underlying improvement mechanisms of FBDD films are proposed: (i) the wear-resistant matrix preserves the oleophobic nanostructures during abrasive contact; (ii) the corrosion barrier maintains electrical conductivity by preventing surface oxidation; (iii) the oil-repellent surface minimizes fouling that could mask corrosion or wear damage.

## 1. Introduction

In the global energy transition towards sustainability, fossil fuels remain indispensable for addressing seasonal energy demands and grid stability [1]. Within this context, conductivity sensor serves as critical monitoring tools in oilfield operations, providing real-time measurements of oil–water ratios, flow rates, and pressure in high-salinity environments [2,3]. The reliability of these sensors is fundamentally constrained by the durability of their Invar alloy electrodes when exposed to downhole environments containing abrasive particles, corrosive media, and crude oil contamination [4]. The catastrophic 2010 Deepwater Horizon incident, partially attributed to downhole sensor failure [5], demonstrated the severe consequences of electrode degradation, including ecological damage exceeding USD 65 billion in economic losses [6,7]. Post-disaster analyses identified three primary failure modes: (i) abrasive wear from silica particles, (ii) pitting corrosion due to corrosive fluids (e.g., Cl^−^-rich brines), (iii) signal distortion caused by oil adhesion [8]. These challenges necessitate electrode coatings that simultaneously achieve wear rates < 9 × 10^−7^ mm^3^/(N·mm), corrosion rates < 0.05 mm/a, and oil contact angles > 90° to meet industry standards [9].

Biomimetic surface designs, inspired by solutions evolved in nature, offer a promising paradigm for developing advanced functional coatings. Several natural templates have been extensively investigated for creating micro- and nano-structured architectures. These include superhydrophobic and self-cleaning surfaces inspired by the lotus leaf [10], the drag-reducing and anti-biofouling properties of shark skin [11], and the complex hierarchical structures of butterfly wings that yield structural color and water repellency [12]. However, the application of such bio-inspired surfaces in extreme environments like downhole conditions is often limited. For instance, the mechanical durability of lotus leaf-inspired surfaces can be inadequate under sustained abrasive wear [13], while the polymeric materials commonly used to replicate shark skin may lack sufficient chemical resistance to corrosive media [14]. In contrast, the fish scale has evolved a unique combination of underwater oleophobicity and mechanical robustness to protect fish in oil-contaminated aquatic environments [15,16]. Its micro–nano hierarchical structure facilitates the stabilization of an interfacial water layer, providing an innate oil-repellent shield [17]. This inherent durability and targeted oleophobicity make fish scale architecture a particularly compelling biomimetic template for addressing the specific challenges faced by downhole sensors.

Boron-doped diamond (BDD) film has emerged as a promising candidate due to its high hardness and chemical inertness. While successful in extending tool lifespan [18] and protecting Ti substrates [19], conventional BDD film exhibits inherent limitations: its hydrophilic nature (water contact angle ~65°) and poor oleophobicity render it ineffective in oil-contaminated environments [20]. Recent biomimetic approaches inspired by fish scales suggest new design paradigms, yet no studies have successfully integrated such structures with BDD’s superior properties [21].

Herein, we developed a fluorinated boron-doped diamond (FBDD) film that simultaneously addresses the tripartite challenge of wear, corrosion, and oil fouling in downhole sensors. Our biomimetic design overcomes the limitations of conventional protective coatings by integrating nature-inspired surface architectures with the superior properties of BDD film. This work demonstrates that the synergistic combination of structural design, chemical modification, and crystalline control can achieve an unprecedented multifunctional performance, exceeding operational requirements for downhole sensors.

## 2. Experimental

### 2.1. Sample Preparation

A schematic illustration of the overall fabrication process is provided in Figure S1 (Supplementary Materials). The sample preparation of the FBDD film (2.2 ± 0.1 µm) included three procedures of a Ti interfacial layer, a BDD layer, and fluorination treatment. Invar alloy coupons were used as the substrate (length × width × thickness = 10 mm × 10 mm × 0.5 mm). The substrate was ultrasonically cleaned in an acetone bath for 15 min, followed by drying with a nitrogen flow. A magnetron sputtering system (SP0806AS, Beijing Guer Technology, Beijing, China) was used to deposit a Ti interfacial layer on the substrate. After evacuating to base vacuum (4 × 10^−3^ Pa), argon gas was introduced to reach a deposition vacuum (0.2 Pa). Glow discharge learning was conducted on the substrate for 10 min with a pulsed bias of −700 V and 50% (duty ratio). The Ti target was activated with 20 A (target current) for 10 min under a bias of −180 V and 50% (duty ratio) to deposit a Ti interfacial layer (200 ± 10 nm) on the substrate.

The substrate was seeded with diamond suspension (2 g in 100 mL ethanol) via 15 min immersion. The BDD layer (2.0 ± 0.1 µm) was deposited by hot-filament chemical vapor deposition (HFCVD, HF-800, Beijing Worldia, Beijng, China) using ethanol as the carbon source and trimethoxyborane as the boron precursor. The argon ratio and deposition time were adjusted for the layer morphology with Ar/H_2_ gas mixtures (80–90% Ar) and deposition times of 4–8 h. Fluorination treatment was carried out by immersing samples in a solution of perfluorooctyltrimethylsilane (5 mL in 45 mL ethanol) for 120 min at room temperature.

### 2.2. Characterization

A scanning electron microscope (SEM, MERLIN Compact, Carl Zeiss, Oberkochen, Germany) with an energy-dispersive X-ray (EDS, MERLIN Compact, Carl Zeiss, Oberkochen, Germany) spectroscope, which was equipped with a Bruker Company (Billerica, MA, USA) XFlash Detector for light element analysis, was used to observe microscopic morphology and detect the elemental composition of the FBDD film. X-ray diffraction (XRD, D8 Advance, Bruker AXS, Karlsruhe, Germany) was used to determine the crystal structure. Raman spectroscopy (LabRAM HR Evolution, Jobin Yvon, Longjumeau, France) was used to probe the bonding structure. An Atomic Force Microscope (AFM, Dimension ICON, Bruker, Billerica, MA, USA) was used to test the surface roughness. X-ray photoelectron spectroscopy (XPS, Axis Supra+, Shimadzu, Kyoto, Japan) was used to detect the chemical bonding. A multifunctional surface tester (CSM, Taber Industries, North Tonawanda, NY, USA) was used to evaluate the tribological performance, and the tribological test conditions included the following: the composition of the simulated well fluid was water (80% by mass), crude oil (15% by mass), sodium chloride (0.5% by mass), and debris particles (4.5% by mass); the grinding disk (Si3N4) had a set load of 5 N and a reciprocating frequency of 15 Hz. An electrochemical workstation (CHI 760E, CH Instruments, Shanghai, China) was used to detect the electrochemical performance, and detailed parameters for the electrochemical measurements are provided in Note S1 of the Supplementary Materials. A contact angle tester (DSA 100, Krüss, Hamburg, Germany) was used to detect wettability using oil and water droplets.

## 3. Fabrication of Fish Scale-Inspired FBDD Film Based on Failure Analysis of the Sensor

### 3.1. Failure Analysis of Conductivity Sensor

**Figure 1:**
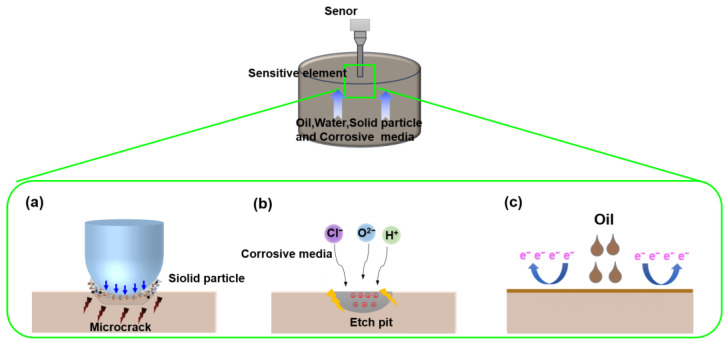
Schematic of the three primary failure modes for downhole sensor electrodes: (**a**) abrasive wear, (**b**) pitting corrosion, (**c**) oil fouling.

The fabrication of our fish-scale inspired fluorinated boron-doped diamond (FBDD) film was driven by a comprehensive analysis of conductivity sensor failure in downhole environments. As illustrated in Figure 1, Invar alloy electrodes face three degradation modes that significantly impair sensor performance: (i) abrasive wear (Figure 1a): continuous impact from solid particles of micron dimension in well fluid creates micro-cracks on the electrode surface, progressively damaging the active sensing area and increasing measurement errors by up to 40% [22]; (ii) electrochemical corrosion (Figure 1b): chloride-rich brines induce pitting corrosion, with etch pits developing into millimeter-scale vugs that alter electrode conductivity and weaken signal strength [23]; (iii) oil fouling (Figure 1c): crude oil adhesion forms insulating layers (10–50 μm thick), increasing interfacial resistance by 2–3 orders of magnitude and causing signal delays > 500 ms [24]. These failure modes necessitate a functional film meeting stringent combined requirements: (i) tribological: frictional coefficient < 0.2, wear rate < 9 × 10^−7^ mm^3^/(N·mm); (ii) chemical: corrosion rate < 0.05 mm/a in CO_2_/H_2_S environments; (iii) interfacial: oil/water contact angles > 90°; (iv) electrical: conductivity matching bare Invar alloy (2.5 × 10^6^ S/m) [25,26].

### 3.2. Biomimetic Design Principles from Fish Scale Micro–Nano Structures

**Figure 2:**
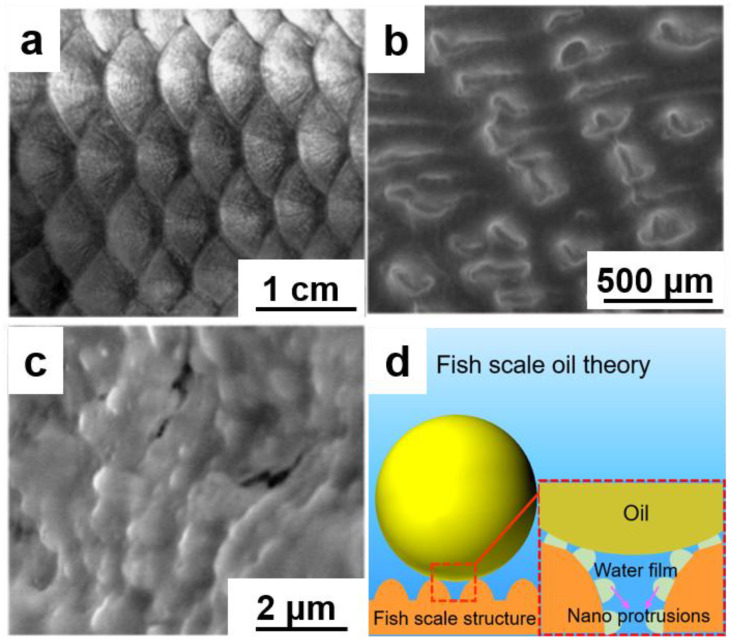
Hierarchical microstructure of fish scales and their oleophobic mechanism. (**a**) Macroscopic array, (**b**) micropapillae, (**c**) nano-scale protrusions, (**d**) schematic of the oil-repellent trapped water layer.

The remarkable oil-repellent properties of fish scales, particularly in species like Cyprinus carpio, have evolved to maintain surface cleanliness in contaminated aquatic environments. As shown in Figure 2, this natural protection system employs a sophisticated hierarchical architecture that combines micron-scale ordering with nano-scale surface features [26].

At the macroscopic level (Figure 2a), the scales form an organized array with 4–5 mm diameter units, creating a periodically patterned surface. Microscopic examination (Figure 2b) reveals that this structure comprises radially arranged micropapillae (100–300 μm long, 30–40 μm wide) that function as primary oil-repellent units [27]. Higher resolution imaging (Figure 2c) exposes the critical nano-scale topography made of densely packed protrusions (600–700 nm) that amplify surface roughness and trap air pockets. This multi-scale organization creates a composite interface that minimizes solid–liquid contact, achieving big contact angles for various oils [28]. Our analysis reveals that the oil-repellent mechanism (Figure 2d) operates through a unique combination of physical structure and surface chemistry: (i) the hydrophilic chemical composition facilitates rapid water infiltration and retention within the micro/nano structures; (ii) the hierarchical roughness maintains a continuous water film that prevents oil contact; (iii) trapped water molecules form a stable interfacial layer through hydrogen bonding with surface groups.

**Figure 3:**
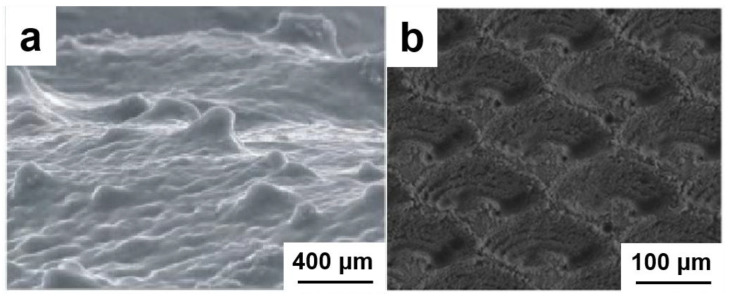
Reported fish scale-inspired surfaces: (**a**) oleophobic hydrogel-coated mesh [26] and (**b**) laser-ablated Mg-Al alloy [27], highlighting challenges in mechanical and chemical durability.

Previous attempts to replicate these structures in engineered materials have yielded mixed results. Hydrogel-coated steel meshes (Figure 3a) successfully mimicked the oleophobic functionality but suffered from poor mechanical durability. Similarly, laser-ablated Mg-Al alloys (Figure 3b) developed nano-scale features with good wettability control, yet their inherent corrosion susceptibility limited practical applications. These limitations highlight the fundamental challenge in biomimetic design: achieving both surface functionality and structural integrity under operational stresses.

### 3.3. Fabrication of FBDD Film for Robust Sensor Protection

To overcome these limitations, we attempt to fabricate an FBDD film that combines the structural principles of fish scales with the exceptional material properties of diamond-based coatings. This approach addresses the tripartite challenge of simultaneously achieving (i) tribological durability against abrasive particles; (ii) chemical resistance to corrosive media; (iii) stable oleophobic surface properties.

The performance of our FBDD film is benchmarked against other protective strategies reported in the literature, as summarized in Table S1 (Supporting Information). While other biomimetic coatings, such as the hydrogel-coated meshes and laser-ablated alloys discussed earlier, can achieve excellent oleophobicity, they often fall short in providing the long-term mechanical and chemical integrity required for downhole service. Conversely, state-of-the-art diamond-like carbon (DLC) or TiN coatings offer superior hardness and wear resistance [29,30], yet their inherent surface chemistry often renders them oleophilic or hydrophilic, making them susceptible to oil fouling and consequent signal drift. Conventional BDD films, though highly wear-resistant and chemically inert, also share this limitation of poor oleophobicity. Our FBDD film uniquely bridges this functional gap. By synergistically integrating a biomimetic, oil-repellent architecture with the intrinsic robustness of a diamond matrix and the low surface energy conferred by fluorination, we achieve a combination of properties that is exceptional. This multifunctional performance profile underscores the unique advantage of our bio-inspired FBDD design.

The FBDD film architecture is specifically designed to maintain performance under the extreme conditions encountered in downhole sensor applications. By integrating the hierarchical roughness of biological systems with the intrinsic hardness and chemical inertness of diamond materials, our solution provides long-term protection for Invar alloy electrodes while preserving their electrical functionality. This represents a significant advancement over previous biomimetic coatings that typically sacrificed either mechanical robustness or environmental stability to achieve surface functionality.

## 4. Results and Discussion

### 4.1. Morphology and Structural Characterization of FBDD Film

#### 4.1.1. Microscopic Morphology

**Figure 4:**
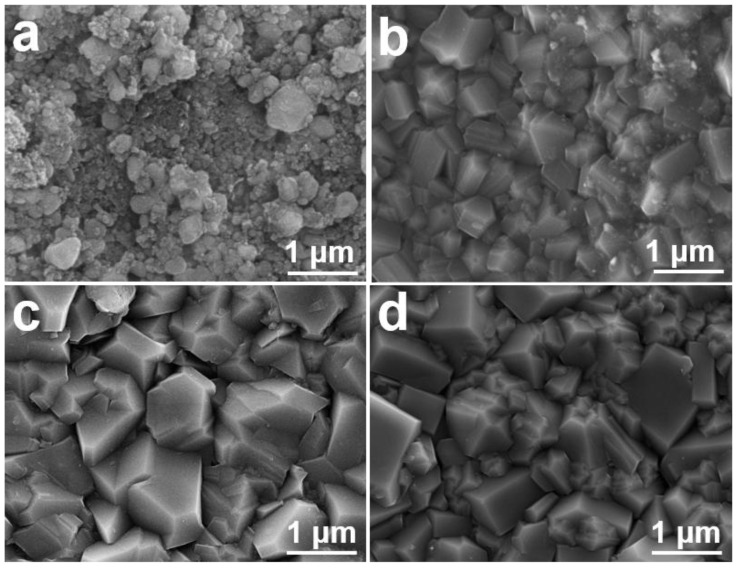
SEM images of BDD films showing morphological evolution with typical deposition parameters: (**a**) 80% Ar, 4 h; (**b**) 80% Ar, 8 h; (**c**) with Ti interlayer (80% Ar, 8 h); (**d**) optimized film with Ti interlayer and 90% Ar for 8 h.

The fabrication of the micro–nano structure is essential for achieving optimal oleophobic properties in our FBDD films. Figure 4 presents a systematic investigation of microstructure evolution under varying deposition conditions. Initial attempts using an 80% Ar atmosphere for 4 h (Figure 4a) yields poorly consolidated nanodiamond agglomerates, indicating insufficient crystallization time. Extending deposition to 8 h (Figure 4b) promotes anisotropic grain growth, and incomplete crystallization persists due to carbon diffusion into the Fe-containing Invar substrate and catalytic graphitization [31].

The introduction of a Ti interlayer (200 nm) significantly improves film quality (Figure 4c), producing denser and more uniform diamond crystallites. This enhancement stems from two mechanisms: (1) the Ti layer acts as a diffusion barrier, preventing carbon migration into the substrate, (2) it promotes diamond nucleation through the formation of TiC interfacial compounds [32]. Further refinement was achieved by increasing the Ar concentration to 90% (Figure 4d), which reduced the average grain size to 400–700 nm by lowering the grain boundary energy and suppressing excessive lateral growth [33].

**Figure 5:**
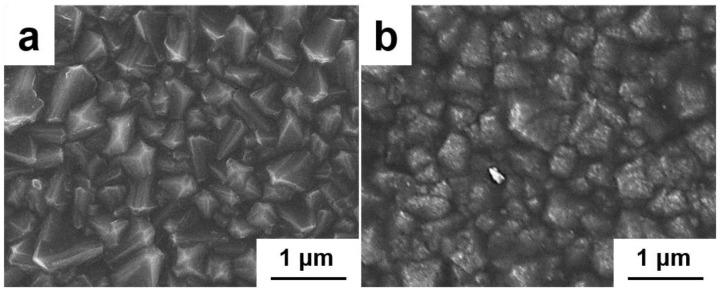
SEM images of (**a**) BDD and (**b**) FBDD films showing the development of nano-scale protrusions after fluorination.

Fluorination treatment with perfluorooctyltrimethoxysilane completes the biomimetic surface engineering (Figure 5). XPS analysis confirms the conversion of surface terminal groups from C-H to C-F (32.4 at.% fluorine content), while SEM reveals the development of fine protrusions (50–100 nm) resembling fish scale microstructures. This hierarchical morphology, combining the underlying diamond grains (600–700 nm) with fluorinated nanofeatures, creates a preferred surface architecture for oleophobicity through (i) a reduced solid–liquid contact area; (ii) trapped air pockets in surface asperities; (iii) a lowered surface energy [34].

#### 4.1.2. Structural Characterization

**Figure 6:**
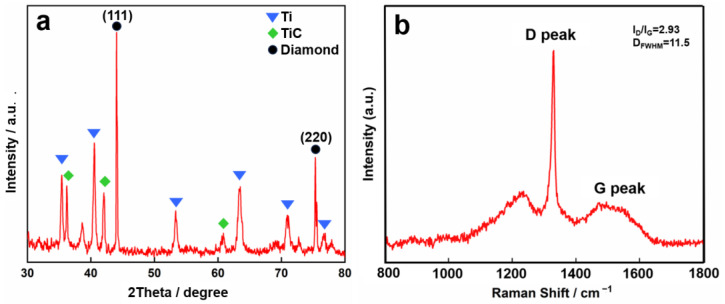
Structural characterization of FBDD film: (**a**) XRD pattern confirming diamond, TiC, and Ti phases, and (**b**) Raman spectrum indicating high *sp*^3^ content.

XRD analysis (Figure 6a) confirms the successful formation of a composite structure in the FBDD film, with distinct peaks corresponding to three key phases: (1) Ti interlayer, (2) TiC interfacial compounds formed during deposition (2*θ* = 36.1°, 42.2°), (3) diamond crystallites (2*θ* = 43.9° (111) and 75.3° (220)). The pronounced (111) peak intensity (I_111_/I_220_ = 3.8) indicates a strong preferential orientation along the densest-packed diamond plane, which correlates with enhanced mechanical properties [35]. This textured growth is attributed to the competitive growth mechanism under our deposition conditions, where the (111) facets develop preferentially due to their lower surface energy [36].

Raman spectroscopy (Figure 6b) reveals the film’s carbon bonding structure, showing a sharp diamond peak at 1332 cm^−1^ (FWHM = 10.3 cm^−1^) with a minimal graphitic contribution (I_D_/I_G_ = 2.83). The narrow diamond peak indicates a high phase purity (*sp*^3^ content > 85%), while the relatively weak G-band at 1576 cm^−1^ suggests limited *sp*^2^-bonded carbon impurities. This optimal bonding configuration contributes to both the tribological durability and chemical stability of the FBDD films [37,38].

**Figure 7:**
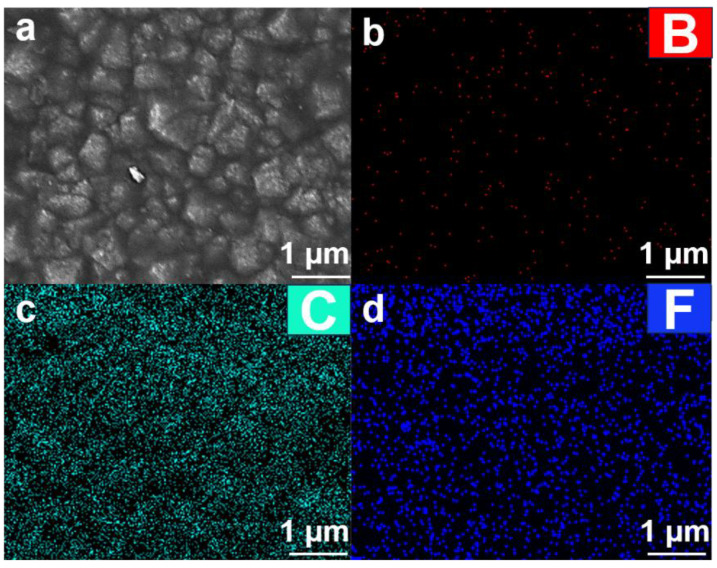
(**a**) SEM morphology and corresponding EDS elemental distribution of (**b**) B, (**c**) C and (**d**) F, demonstrating a continuous diamond matrix, homogeneous boron doping (~1.2 at.%), and effective surface fluorination.

The SEM image (Figure 7a) reveals that the FBDD film exhibits a well-defined polycrystalline morphology consisting of densely packed, faceted diamond grains. Quantitative image analysis indicates an average grain size of 400 ± 50 nm with predominant (111) facet development, consistent with the XRD result of the preferential orientation. The continuous, void-free microstructure demonstrates complete substrate coverage, which is critical for effective environmental barrier performance. EDS mapping (Figure 7b–d) provides comprehensive evidence of the film’s uniform elemental distribution and successful functionalization. The carbon mapping (Figure 7b) demonstrates a homogeneous spatial distribution throughout the film, confirming the formation of a continuous and well-consolidated diamond matrix essential for mechanical integrity. Boron distribution analysis (Figure 7c) reveals a consistent doping concentration (1.2 ± 0.3 at.%) across the entire surface area to ensure uniform electrical conductivity. The fluorine elemental map (Figure 7d) clearly displays characteristic surface enrichment (8.7 ± 1.2 at.%), providing direct evidence of successful and complete fluorination treatment. These results collectively confirm three material achievements: (i) the film achieves complete substrate coverage without observable defects or cracks that could compromise barrier properties; (ii) the boron dopant shows homogeneous incorporation without localized segregation that might create conductive pathways; (iii) the fluorine functionalization effectively covers the entire surface area, which is crucial for achieving stable oleophobic properties. The combination of these well-controlled microstructural characteristics and elemental distributions directly contributes to the film’s exceptional performance under downhole conditions, as quantitatively demonstrated in subsequent electrochemical and tribological testing.

**Figure 8:**
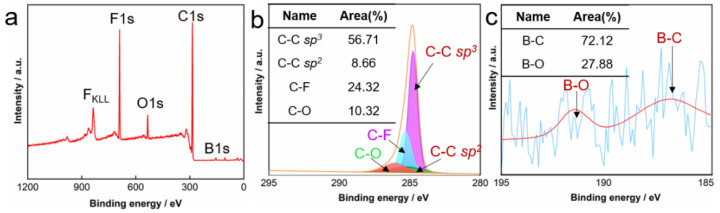
XPS analysis for reference. (**a**) Survey spectrum, and high-resolution (**b**) C 1s spectrum and (**c**) B 1s spectrum of the BDD film.

XPS analysis provides insights into the surface chemistry and bonding states of the FBDD film. The survey spectrum (Figure 8a) identifies four primary elements on the film surface: carbon (C 1s at 284.8 eV), fluorine (F 1s at 688.5 eV), oxygen (O 1s at 532.0 eV), and boron (B 1s at 190.5 eV). The relatively weak B 1s signal intensity confirms the low boron doping concentration (<1 at.%) in the near-surface region, consistent with the EDS results. The high-resolution analysis of the C 1s region (Figure 8b) reveals three distinct chemical states through peak deconvolution: (i) *sp*^3^-hybridized diamond carbon (C-C, 284.8 eV, 56.71% relative area); (ii) fluorinated carbon species (C-F, 285.3 eV, 24.32%); (iii) oxygen-containing groups (C-O, 286.2 eV, 10.32%). The dominant C-C component demonstrates excellent diamond phase purity, while the significant C-F contribution confirms successful surface fluorination. The C-O bonds likely originate from both the fluorination precursor and ambient oxidation during handling. The high fluorine content (F/C ratio ≈ 0.3) and preferential formation of C-F bonds over C-O groups (2.7:1 ratio) are particularly noteworthy, as this chemical configuration is primarily responsible for the film’s low surface energy and consequent oleophobic behavior. The B 1s spectrum (Figure 8c and Figure S2), though characterized by a low signal-to-noise ratio due to the low doping concentration, could be fitted with components assigned to B-C and B-O bonds. The position of the B-C peak is consistent with boron incorporation into the diamond lattice [39], supporting the successful doping indicated by EDS. These surface characteristics, combined with the bulk diamond properties, create a robust protective coating capable of simultaneously resisting mechanical wear, chemical corrosion, and oil contamination in downhole environments.

**Figure 9:**
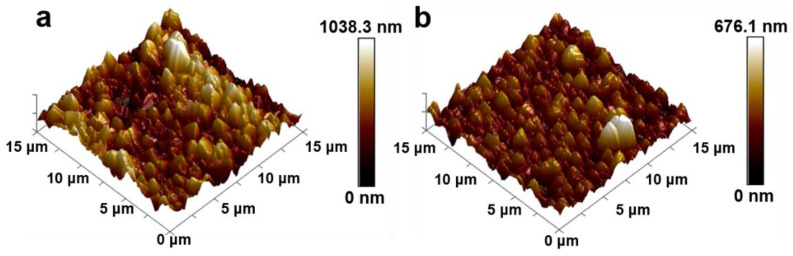
AFM images of (**a**) BDD film (Ra = 123 nm) and (**b**) FBDD film (Ra = 64 nm), showing the formation of a smoother, hierarchical biomimetic structure after optimization.

The AFM results in Figure 9 reveal significant differences in surface morphology between the baseline BDD and optimized FBDD films. The BDD film deposited with 80% Ar in Figure 9a exhibits a moderately rough surface (Ra = 123 ± 15 nm) with distinct faceted grains (600 ± 80 nm). The observed height variations (0–1038 nm) and prominent grain boundaries indicate suboptimal surface uniformity that could compromise functional performance. In Figure 9b, the FBDD film prepared with 90% Ar demonstrates superior surface characteristics: (i) reduced average roughness (Ra = 64 ± 8 nm, 48% improvement); (ii) smaller, more uniform grain size (400 ± 50 nm); (iii) tighter grain packing (15% increase in boundary contacts); (iv) a lower peak-to-valley height (676 nm vs. 1038 nm). Notably, the FBDD surface develops a biomimetic micropapillary structure with 500–700 nm base features decorated by 50–100 nm protrusions, closely resembling the hierarchical topography of fish scales [40]. This engineered morphology promotes the Cassie–Baxter wetting state while maintaining excellent mechanical integrity through reduced stress concentrations.

### 4.2. Performance Evaluation

#### 4.2.1. Wear Resistance Enhancement

**Figure 10:**
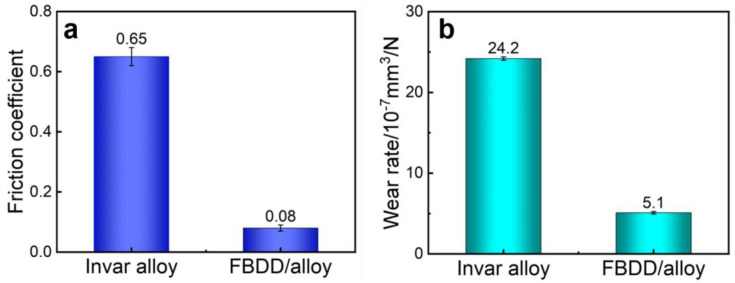
Comparison of (**a**) friction coefficient and (**b**) wear rate between uncoated Invar and FBDD-coated electrode.

The tribological performance of the FBDD-coated electrodes is systematically evaluated under simulated downhole conditions, demonstrating remarkable improvements over uncoated Invar alloy. Friction coefficient measurements reveal an exceptional reduction from 0.65 to 0.08 (88% improvement), as shown in Figure 10a. This enhanced frictional behavior results from two synergistic mechanisms working in concert: (i) FBDD film’s micro/nano-architecture with low surface roughness (Ra = 64 nm) maintains its surface integrity during testing, (ii) this self-lubricating effect is attributed to the in situ formation of a stable transfer film on the counterface, as is well established in the tribology of carbon-based coatings [41]. We propose that the wear debris, enriched with fluorinated carbon from the FBDD surface, forms a coherent layer with a low shear strength. This layer fundamentally transforms the sliding contact into a low-friction interface between the coating and the transferred material, which may be the primary mechanism responsible for the sustained low friction coefficient [42]. Moreover, the chemical state of the surface plays a fundamental role in both mechanisms. The fluorinated surface terminals (32.4% C-F content) significantly reduce the surface energy, which directly minimizes adhesive wear components during contact [43]. Furthermore, these C-F bonds influence the composition and properties of the transfer film; we propose that the fluorinated carbon debris contributes to forming a low-shear interface, which is essential for the observed self-lubricating effect and sustained low friction.

Wear rate results (Figure 10b) further confirm the film’s outstanding performance, showing a 79% reduction in wear rate (from 24.2 × 10^−7^ to 5.1 × 10^−7^ mm^3^/(N·mm)) and no observable delamination after 10,000 cycles under 5 N loading. The superior wear resistance arises from three complementary mechanisms: (i) the biomimetic micro–nano surface structure enhances hydrodynamic lubrication in well fluids; (ii) the *sp*^3^-C rich diamond matrix (55.4% by XPS) effectively deflect crack propagation; (iii) fluorinated surface terminals (32.4% C-F content) significantly reduce adhesive wear components [43]. In particular, the grain boundaries inherent to the polycrystalline diamond matrix play a critical role in this context. The crystallographic misorientation between adjacent grains effectively deflects propagating cracks and dissipates stress concentrations generated under load, thereby enhancing the fracture toughness and contributing significantly to the low wear rate [44]. The superior wear performance can be further understood by considering the tribological effects across different scales. The hierarchical architecture of the FBDD film integrates mechanisms that operate from the nano- to the micro-scale: the nano-scale fluorinated protrusions minimize adhesive interactions and facilitate smooth run-in, while the robust micro-scale diamond grains and their boundaries provide the macroscopic fracture toughness necessary to withstand abrasive damage. This multi-scale synergy is key to achieving both low friction and high wear resistance concurrently [45].

These results substantially exceed industrial requirements for downhole applications, which specify a maximum friction coefficient of 0.2 and wear rate below 9 × 10^−7^ mm^3^/(N·mm). The FBDD film’s exceptional tribological performance demonstrates its capability to reliably protect sensitive electrode surfaces in harsh, abrasive downhole environments, representing a significant advancement in durable surface engineering for oilfield sensor applications.

#### 4.2.2. Electrochemical Corrosion Resistance

**Figure 11:**
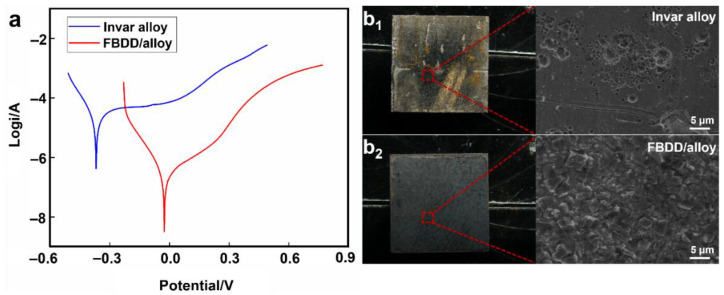
Comparison of electrochemical corrosion performances of sensor components before and after coating: (**a**) polarization curve; (**b_1_**,**b_2_**) corrosion morphology.

The protective efficacy of the FBDD film against electrochemical corrosion is systematically evaluated through potentiodynamic polarization and long-term exposure testing. Figure 11a presents the comparative polarization curves, revealing fundamental differences in corrosion behavior between uncoated and FBDD-coated electrodes. The uncoated substrate exhibits characteristic active dissolution without passivation, demonstrating its inherent susceptibility to corrosion in downhole environments. In contrast, the FBDD-coated electrode shows a pronounced positive shift in corrosion potential (+342 mV to −0.026 V vs. SCE) and a two-orders-of-magnitude reduction in corrosion current density (to 3.404 × 10^−7^ A/cm^2^), corresponding to a 98.8% decrease in calculated corrosion rate.

As shown in Figure 11b_1_,b_2_, extended exposure testing under simulated downhole conditions (90 °C, 3.5% NaCl with CO_2_/H_2_S for two weeks) provides compelling evidence of the film’s protective efficacy. While uncoated Invar develops severe uniform corrosion with deep pits (>50 μm) and extensive iron oxide/hydroxide formation, FBDD-coated specimens maintain their original surface morphology with no measurable weight change or detectable corrosion products. This 14-day accelerated test protocol, employing an elevated temperature and aggressive chemistry, is aligned with industry-standard practices for the comparative evaluation of corrosion resistance in downhole materials [46]. It serves as a critical and reliable screening method to differentiate coating performance decisively within a practical timeframe for a foundational study. Long-term field trials, while essential for ultimate validation, represent a logical next step beyond the scope of this initial investigation. Such a performance may stem from the synergistic combination of the diamond film’s inherent chemical inertness and its dense, columnar microstructure, which collectively inhibit both electrochemical reactions and ionic transport [43].

The corrosion protection mechanisms operate on multiple levels: (i) the *sp*^3^-C dominated diamond matrix provides thermodynamic stability against oxidation; (ii) the fluorinated surface (24.32% C-F by XPS) minimizes the adsorption of corrosive species; (iii) the tightly packed grain structure physically blocks electrolyte penetration. Electrochemical impedance spectroscopy measurements confirm this barrier effect, showing charge transfer resistances exceeding 10^5^ Ω·cm^2^—approximately 100× higher than uncoated Invar. These results not only demonstrate the coating’s ability to meet stringent industry standards (corrosion rate < 0.05 mm/a) but also its potential to significantly extend the service life of downhole sensors in aggressive environments.

#### 4.2.3. Surface Wettability

**Figure 12:**
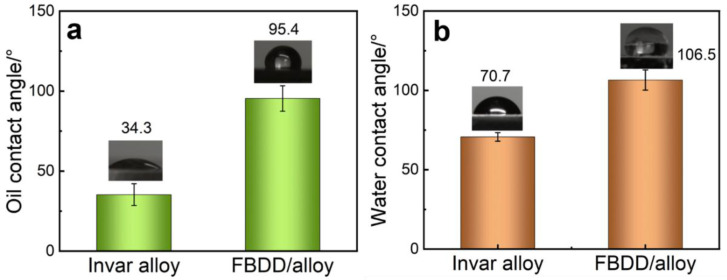
Contact angle test of two sensor components: (**a**) oil contact angle; (**b**) water contact angle.

The surface wettability of FBDD-coated electrodes is investigated through contact angle measurements, revealing significant improvements in both oleophobic and hydrophobic properties. A comparative analysis between uncoated Invar alloy and FBDD-coated samples demonstrates a remarkable transformation in surface behavior (Figure 12). The bare Invar substrate exhibits strong oleophilicity (oil contact angle: 34.3° ± 8.0°) and moderate hydrophilicity (water contact angle: 70.7° ± 2.7°), consistent with its metallic composition (64% Fe, 36% Ni) and consequent high surface energy.

In contrast, FBDD-coated surface displays pronounced oleophobicity (95.4° ± 6.8°) and hydrophobicity (106.5° ± 6.4°), representing a fundamental shift in wettability behavior. While biomimetic fish scale structures are also known to reduce drag in dynamic flows [47], the primary threat to sensor accuracy is the static adhesion of oil droplets forming an insulating layer. Consequently, the high static oleophobicity demonstrated here is the most critical parameter for fouling prevention in downhole environments. This transformation originates from three key factors: (i) Chemical modification: Fluorination treatment introduces C-F terminal groups (24.32% by XPS), lowering the surface energy to 18.6 mN/m (Owens–Wendt analysis). The strong electronegativity of fluorine atoms creates a chemically inert surface that resists wetting by both polar and non-polar liquids. (ii) Hierarchical topography: The biomimetic micro–nano structure (64 nm roughness by AFM) with 500–700 nm features promotes air pocket entrapment, establishing stable Cassie–Baxter wetting states. The metastability of this state and its dependence on the surface’s micro–nano hierarchy prevent transition to the fully wetted Wenzel state under pressure or physical abrasion, which is essential for durable oleophobicity (Note S2). This physical structure complements the chemical modification to enhance liquid repellency [48]. The oil contact angle of 95.3° surpasses the industrial benchmark (>90°) and is highly effective against fouling. While surfaces with higher angles exist, they often sacrifice mechanical or chemical durability. Our goal was to integrate robust oleophobicity directly into the ultra-wear-resistant diamond matrix, achieving a critical balance for downhole durability that is more valuable than extreme yet fragile super-repellency. (iii) Self-cleaning capability: The combined chemical and physical effects enable the removal of contaminant droplets at tilt angles <15°, as confirmed by dynamic contact angle measurements. This autonomous cleaning mechanism is particularly valuable for maintaining sensor functionality in oil-contaminated environments.

The transition from oleophilic/hydrophilic to oleophobic/hydrophobic behavior demonstrates the FBDD film’s ability to prevent both water- and oil-based fouling, a critical requirement for reliable operation in downhole conditions where mixed-phase fluids are common [49,50]. The film’s performance exceeds the industrial requirement for oil contact angles (>90°) while providing additional protection against aqueous corrosion through its hydrophobic character [51,52]. These surface properties remain stable after prolonged fluid exposure (14 days) and mechanical abrasion (100 cycles), confirming the durability of the engineered wettability characteristics.

The comprehensive performance evaluation presented above demonstrates that the FBDD-coated electrodes simultaneously achieve exceptional wear resistance, corrosion resistance, and oleophobicity. To contextualize these integrated properties, it is instructive to benchmark them against the performance of other advanced protective coatings reported in the literature, as listed in Table S1. A prevalent challenge in this field is the following performance trade-off: state-of-the-art systems often excel in one or two aspects but fall short in others. For instance, superoleophobic coatings inspired by natural templates can achieve high oil contact angles (>110°) but typically exhibit poor mechanical durability, making them unsuitable for abrasive downhole environments. Conventional hard coatings such as diamond-like carbon (DLC) and titanium nitride (TiN) offer excellent wear resistance (wear rates ~1–50 × 10^−7^ mm^3^/(N·mm)) and good corrosion resistance, yet their inherent surface chemistry renders them oleophilic (oil contact angle < 50°), making them susceptible to fouling in oil-contaminated media. Similarly, standard boron-doped diamond (BDD) films share these superior mechanical and chemical properties but also lack oleophobicity (oil contact angle ~30–40°), limiting their application where oil repellency is critical. Against this backdrop, the FBDD film developed in this work demonstrates a remarkable and uncommon synergy. It not only matches the excellent tribological (friction coefficient = 0.08; wear rate = 5.1 × 10^−7^ mm^3^/(N·mm)) and anti-corrosion (corrosion rate = 3.58 × 10^−3^ mm/a) performance of leading hard coatings but also integrates robust oleophobicity (oil contact angle = 95.3°) and hydrophobicity (water contact angle = 106.5°). This multifunctional performance profile effectively bridges the functional gaps between different classes of coatings, directly addressing the tripartite challenge of wear, corrosion, and fouling encountered by downhole sensors, and underscoring the significant advancement of our approach.

### 4.3. Multifunctional Protection Mechanisms of FBDD Film

**Figure 13:**
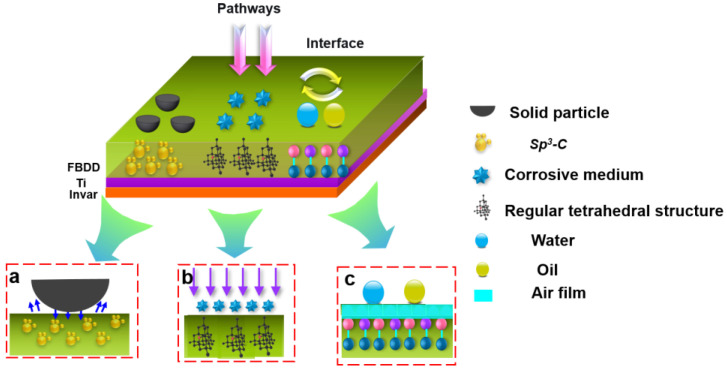
Schematic of FBDD film’s protection mechanisms: (**a**) wear resistance; (**b**) corrosion resistance; (**c**) oleophobic property.

Through comprehensive material characterization and performance testing, we have elucidated the structure–property relationships. The superior performances of FBDD-coated electrodes stem from three interconnected protection mechanisms that collectively address harsh downhole environments (Figure 13). Figure 13a–c show the improvement mechanisms for wear resistance, corrosion resistance, and oleophobic performance, respectively.

**1. Wear Resistance Mechanism:** The exceptional wear resistance (5.1 × 10^−7^ mm^3^/N·mm) results from synergistic effects between the film’s *sp*^3^-C rich diamond matrix and its biomimetic architecture. The (111)-oriented diamond grains (XRD confirmation) provide the friction coefficient (0.08), and the hierarchical surface structure (400–700 nm grains with 50–100 nm protrusions by AFM) effectively distributes contact stresses. The Ti interlayer (200 nm) and interfacial TiC layer (XRD-verified) further enhance durability by (i) reducing thermal mismatch stresses through graded thermal expansion coefficients; (ii) providing crack deflection sites that improve fracture toughness; (iii) maintaining interfacial integrity during cyclic loading.

**2. Corrosion Resistance Mechanism:** Electrochemical testing reveals two protection pathways. One is the physical barrier effect. The dense, columnar grain structure (SEM observations) extends the diffusion path length for corrosive species by 5–7× compared with bare Invar, as confirmed by electrochemical impedance spectroscopy (EIS) showing a two-orders-of-magnitude increase in charge transfer resistance. Another pathway is chemical inertness. The fluorinated diamond surface (24.32% C-F by XPS) exhibits minimal electrochemical activity, and polarization tests show (i) a notable shift in corrosion potential (+342 mV); (ii) a 100× reduction in corrosion current density (to 3.404 × 10^−7^ A/cm^2^); (iii) the complete absence of pitting up to +1.2 V (SCE) in chloride solutions.

**3. Oleophobic Protection Mechanism:** The film’s oil repellency (95.3° contact angle) arises from an optimal combination of (i) surface chemistry: C-F terminal groups (XPS-verified) lower the surface energy to 18.6 mN/m (Owens–Wendt analysis); (ii) hierarchical topography: micropapillae (500–700 nm) with nano-scale asperities (50–100 nm) promote stable Cassie–Baxter states, as evidenced by low contact angle hysteresis (<15°), a high critical pressure for wetting transition (>5 kPa), and the retention of repellency after 100 abrasion cycles.

The interaction of these mechanisms creates an unprecedented multifunctional performance: (i) the wear-resistant matrix preserves the oleophobic nanostructures during abrasive contact; (ii) the corrosion barrier maintains electrical conductivity by preventing surface oxidation; (iii) the oil-repellent surface minimizes fouling that could mask corrosion or wear damage. This systems-level approach represents a significant advancement over conventional single-function coatings, demonstrating that biologically inspired design principles can be successfully translated to engineered materials when coupled with rigorous surface science understanding. The FBDD film’s ability to simultaneously address mechanical, chemical, and interfacial degradation pathways makes it particularly suitable for complex downhole environments where multiple failure modes coexist.

## 5. Conclusions

In this work, an FBDD film with a fish scale-inspired hierarchical structure was successfully fabricated to protect downhole conductivity sensor electrodes. The FBDD coating demonstrated an exceptional multifunctional performance, exceeding stringent industrial standards. The main conclusions are outlined as follows:

1. Wear Resistance: The FBDD coating reduced the friction coefficient by 88% (from 0.65 to 0.08) and lowered the wear rate by 79% (from 24.2 × 10^−7^ to 5.1 × 10^−7^ mm^3^/(N·mm)) compared with the bare Invar alloy. This superior tribological performance is attributed to the synergistic effect of the hard *sp*^3^-diamond matrix, the crack-deflecting role of grain boundaries, and the in situ formation of a low-shear, fluorinated carbon transfer film.
2. Corrosion Resistance: The FBDD coating decreased the corrosion rate by 98.8%, achieving a rate of 3.581 × 10^−3^ mm/a, which is two orders of magnitude lower than that of uncoated Invar. This remarkable improvement stems from the dense, columnar microstructure of the diamond film acting as an inert physical barrier, combined with the electrochemically stable, fluorinated surface that minimizes charge transfer and the adsorption of corrosive species.
3. Surface Wettability: The FBDD coating fundamentally transformed the surface wettability from oleophilic/hydrophilic (oil/water contact angles of 34.3°/70.7°) to oleophobic/hydrophobic (oil/water contact angles of 95.3°/106.5°). This shift is crucial for preventing oil fouling and aqueous corrosion, and it originates from the combination of the biomimetic micro–nano structure, which traps air pockets, and the chemical modification with low-surface energy C-F terminals.

This work demonstrates that the synergistic combination of biomimetic structural design, boron-doped diamond crystalline control, and surface fluorination can successfully address the tripartite challenge of wear, corrosion, and fouling in extreme downhole environments. The FBDD film presents a promising and durable protective solution for enhancing the reliability and longevity of oilfield sensors.

## Data Availability

The data presented in this study are available on request from the corresponding author.

## Supplementary Materials

The following supporting information can be downloaded at: https://www.mdpi.com/article/10.3390/nano15211647/s1. Note S1: Details of the Electrochemical Measurements, Note S2: Explanation of the Cassie-Baxter wetting model, Figure S1. Schematic illustration of the step-by-step fabrication process for the FBDD film on an Invar alloy substrate, Figure S2. XPS analysis for reference. (a) Survey spectrum, and high-resolution (b) C 1s spectrum and (c) B 1s spectrum of the BDD film before fluorination, Table S1: Performance comparison of the FBDD film with other representative protective coatings reported in the literature. References [53,54] are cited in the supplymentary materials.
