# Supplementary material for: Inspired Fluorinated BDD Film for Multifunctional Protection of Downhole Sensor Electrodes
Source: Nanomaterials (Basel). 2025 Oct 28;15(21):1647. doi: 10.3390/nano15211647 (PMC12609872; doi:10.3390/nano15211647)
Supplement: Supplementary file 1 [file nanomaterials-15-01647-s001.zip › nanomaterials-3897538-supplementary.pdf]

nanomaterials

Supplementary Materials

Inspired Fluorinated BDD Film for Multifunctional Protection of Downhole Sensor Electrodes

Jiahao Liu [1], Shuo Zhao [2,3], Jincan Wang [2], Jiaxi Liu [2], Xiang Yu [2,*] and Jing Zhang [4,*]

1 Programme of Sustainable Energy Technology and Management, Faculty of Science and Technology, Beijing Normal-Hong Kong Baptist University, Zhuhai 519087, China; v530206022@mail.uic.edu.cn
2 Engineering Research Center, Ministry of Education for Geological Carbon Storage and Low Carbon Utilization of Resources, Beijing Key Laboratory of Materials Utilization of Nonmetallic Minerals and Solid Wastes, School of Materials Science and Technology, China University of Geosciences (Beijing), Beijing 100083, China; shuozhao@buaa.edu.cn (S.Z.); 2103240085@email.cugb.edu.cn (J.W.); 2003250034@email.cugb.edu.cn (J.L.)
3 Institute of Atomic Manufacturing, Beihang University, Beijing 100191, China
4 Shandong Provincial Key Laboratory of High Strength Lightweight Metallic Materials, Advanced Materials Institute, Qilu University of Technology (Shandong Academy of Sciences), Jinan 250014, China
* Correspondence: yuxiang@cugb.edu.cn (X.Y.); jingzhang@qlu.edu.cn (J.Z.)

Note S1: Details of the Electrochemical Measurements

(Cited in Section 2.2)
The electrochemical corrosion performance was evaluated using an electrochemical workstation (CHI 760E, CH Instruments, China). The tests were conducted using a standard three-electrode electrochemical cell configuration. Main parameters were as follows: Electrolyte: 3.5 wt% sodium chloride (NaCl) solution, saturated with a mixed gas of carbon dioxide (CO_2) and hydrogen sulfide (H_2S).Temperature: 90 °C, maintained using a thermostatically controlled water bath. Working Electrode: The FBDD-coated or uncoated Invar alloy sample, with an exposed surface area of 1.0 cm². Counter Electrode: A platinum (Pt) plate. Reference Electrode: A saturated calomel electrode (SCE). Test Procedure: The open circuit potential (OCP) was monitored until it stabilized (typically for 30 minutes). Potentiodynamic polarization tests were then performed from -0.25 V to +0.25 V versus the OCP at a scan rate of 1 mV/s.

Note S2: (Cited in Section 4.2.3)

Note S2: Explanation of the Cassie-Baxter wetting model.
The exceptional oleophobicity observed in this work can be described by the Cassie-Baxter wetting model [7]. This model applies to heterogeneous surfaces where a liquid droplet sits on a composite interface of solid and air.
The fundamental principle is that the apparent contact angle (θ*) on a rough surface is amplified according to the following equation:

$$Cos\theta *= f_s \, cos\theta \; - \; f_v$$

Where: θ* is the apparent contact angle observed on the rough surface; θ is the intrinsic contact angle on a flat surface of the same material; f_s is the fractional area of the solid-liquid contact; f_v is the fractional area of the liquid-vapor (air) contact ($f_s + f_v = 1$).
A key peculiarity of the Cassie-Baxter state is its metastability. The stability of this air-trapped state is not guaranteed and depends critically on the surface's hierarchical micro-nano structure. The multiscale roughness of our FBDD film, inspired by fish scales, provides sufficient energy barriers to prevent the collapse of the air pockets and the transition to the fully wetted (Wenzel) state, even under some physical perturbation. This robustness is crucial for achieving the durable oleophobic performance required in downhole environments.

Figure S1 (Cited in Section 2.1)

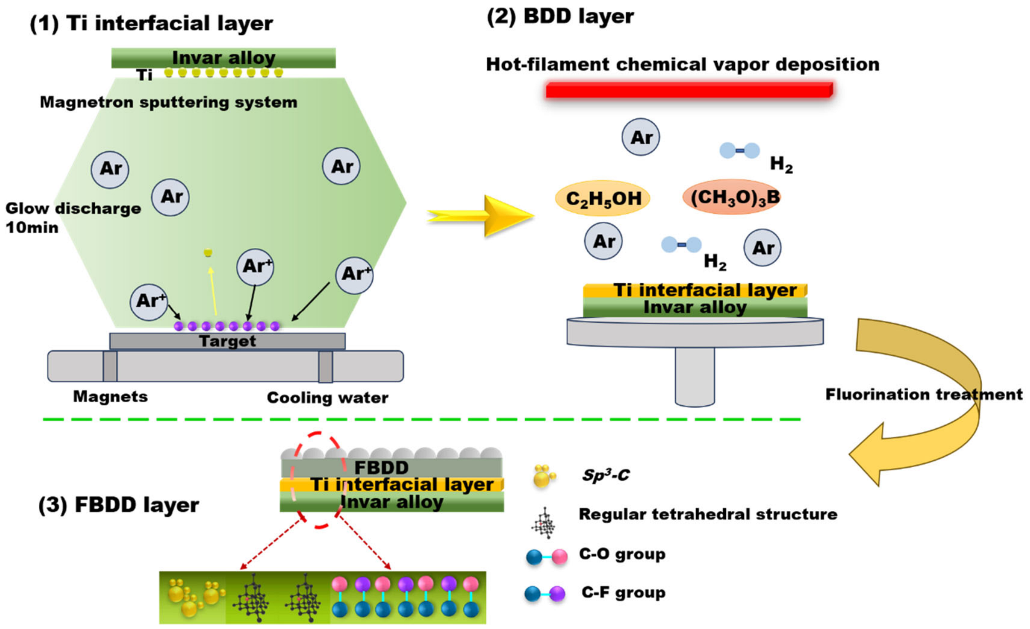

Figure S1. Schematic illustration of the step-by-step fabrication process for the FBDD film on an Invar alloy substrate

Figure S2 (Cited in Section 4.1.2)

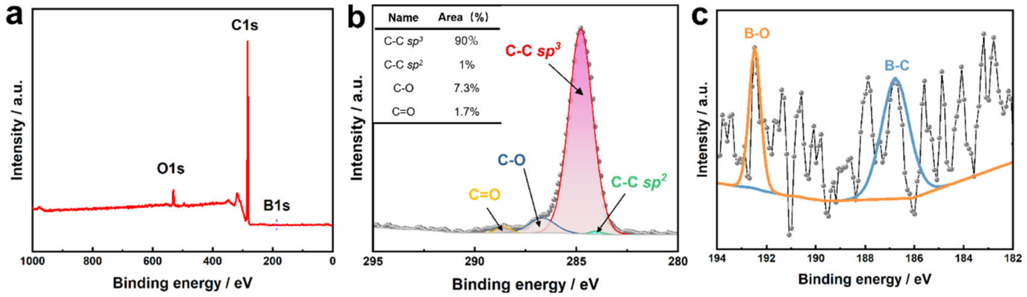

Figure S2. XPS analysis for reference. (a) Survey spectrum, and high-resolution (b) C 1s spectrum and (c) B 1s spectrum of the BDD film before fluorination.

Table S1: (Cited in Section 3.3 and 4.2)

Table S1: Performance comparison of the FBDD film with other representative protective coatings reported in the literature.

Coating Type Material	Friction Coefficient	Wear Rate (10^{-7} mm^3/(N·mm))	Corrosion Rate (mm/a)	Oil Contact Angle (°)	Key Limitations	Ref.
This Work: FBDD on Invar	0.08	5.1	3.58×10^{-3}}	95.3	None identified	
Hydrogel-coated Mesh (Biomimetic)	~0.3-0.5	> 1000 (severe)	N/A (Polymer degrades)	> 120	Poor Mechanical Durability, Swells/Degrades	[1]
Laser-ablated Mg-Al Alloy (Biomimetic)	N/A	N/A	> 0.1 (High)	> 110	Inherently Low Corrosion Resistance	[2]
Diamond-Like Carbon (DLC)	0.05-0.15	1 - 10	~10^{-4} - 10^{-3}	< 30 Oleophilic	High residual Stress, Poor Oleophobicity	[3, 4]
TiN Coating	0.4-0.6	10 - 50	~10^{-3}	< 50 Hydrophilic	Relatively High Friction, Poor Oleophobicity	[3]
Conventional BDD Film	0.1-0.2	2 - 20	~10^{-4} - 10^{-3}	~30-40 Oleophilic	Poor Oleophobicity leads to Fouling	[5]
Fluorinated Polymer Coating	0.2-0.5	> 100 (Soft)	N/A (Polymer)	> 100	Low Wear Resistance, Soft	[6]

Reference

1. Jinlong, Z.; Huili, L.; Juan, D.; Wenchen, R.; Pingping, G.; Shimei, X.; Jide, W. A Robust and Coarse Surface Mesh Modified by Interpenetrating Polymer Network Hydrogel for Oil-Water Separation. *J. Appl. Polym. Sci.* **2015**, 132, 41949.
2. Wanting, R.; Haifeng, Z.; Zhigang, M.; Liang, C.; Xiaowei, L. Stable Drag Reduction of Anisotropic Superhydrophobic/Hydrophilic Surfaces Containing Bioinspired Micro/Nanostructured Arrays by Laser Ablation. *Colloids Surf. A* **2021**, 622, 126712.
3. Ciarsolo I., Fernández X. Tribological comparison of different C-based coatings in lubricated and unlubricated conditions. *Surf. Coat. Technol.* **2014**, 257, 278–285.
4. Shi B., Wu Y. A review on diamond-like carbon-based films for space tribology. *Mater. Sci. Technol.* **2022**, 38, 1151–1167.
5. Cai, Y.; Li, J.; Yi, L.; Yan, X.; Li, J. Fabricating Superhydrophobic and Oleophobic Surface with Silica Nanoparticles Modified by Silanes and Environment-Friendly Fluorinated Chemicals. *Appl. Surf. Sci.* **2018**, 450, 102–111.
6. Kredel J., Schmitt D. Cross-Linking Strategies for Fluorine-Containing Polymer Coatings for Durable Resistant Water- and Oil-Repellency. *Polymers* **2021**, 13, 723.
7. Wang Y. and Gong X. Special oleophobic and hydrophilic surfaces: approaches, mechanisms, and applications. *J. Mater. Chem. A* **2017**, 5, 3759–3773.
